# Connecting to motor recovery after stroke

**DOI:** 10.1093/braincomms/fcaa067

**Published:** 2020-05-06

**Authors:** Jill H Fowler, Raj N Kalaria

**Affiliations:** f1 Centre for Discovery Brain Science, University of Edinburgh, Edinburgh EH16 4SB, UK; f2 Translational and Clinical Research Institute, Newcastle University, Newcastle Upon Tyne NE4 5PL, UK

## Abstract

This scientific commentary refers to ‘Structural brain networks and functional motor outcome after stroke –a prospective cohort study’, by Schlemm *et al.* (https://doi.org/10.1093/braincomms/fcaa001) in *Brain Communications* and ‘Brain responsivity provides an individual readout for motor recovery after stroke’ by Tscherpel *et al.* (https://doi.org/10.1093/brain/awaa127) in *Brain*

**This scientific commentary refers to ‘Structural brain networks and functional motor outcome after stroke –a prospective cohort study’, by Schlemm *et al.* (https://doi.org/10.1093/braincomms/fcaa001) in *Brain Communications* and ‘Brain responsivity provides an individual readout for motor recovery after stroke’ by Tscherpel *et al.* (https://doi.org/10.1093/brain/awaa127) in *Brain***

Although stroke is the second leading cause of death worldwide, mortality due to stroke has declined in recent decades. This is explained by the reduced incidence of stroke combined with improved treatment and management of strokes (GBD 2016 Neurology Collaborators, 2019). Due to increasing survival rates after ischaemic stroke (Waziry *et al.*, 2020), there is an expanding population of patients living with long-term consequences of stroke, including persistent motor and cognitive deficits. While there is high variability in recovery due to age, gender, behavioural experiences and genetics, there can be remarkable recovery post-stroke (Cramer, 2008). With an increasingly ageing population, the total numbers of strokes are predicted to rise, with one study forecasting a doubling in the number of strokes by 2050 (Gorelick, 2019). Furthermore, the risk of dementia after stroke is high, particularly after a severe stroke (Allan *et al.*, 2011; Pendlebury *et al.*, 2019). Therefore, it is important to understand more about the long-term structural and functional changes that occur in the brain subsequent to stroke that may promote recovery or conversely impede recovery and the mechanisms that lead to additional neurological sequelae such as dementia. Two recent papers in *Brain Communications* (Schlemm *et al.*, 2020) and *Brain* (Tscherpel *et al.*, 2020) contribute to our understanding of chronic structural and functional changes after stroke.

The original term ‘connectome’ refers to the network of anatomical connections between neural elements of the brain (Sporns *et al.*, 2005). Since its definition 15 years ago, the phrase ‘connectome’ has been generalized to include anatomical connectivity (as investigated using non-invasive diffusion-weighted MRI imaging) as well as functional connectivity (investigated with functional MRI, EEG and magnetoencephalography). Connectome studies have highlighted the fact that normal neurological function is dependent on not only the structural and functional integrity of grey matter regions and their white matter connections, but also the balanced interplay of activity between multiple interconnected brain regions. The damage caused by an ischaemic stroke to the brain is often defined by the focal area of necrosis that occurs quickly after the onset of stroke and the surrounding hypoperfused ‘penumbral’ region. However, it is also recognized that stroke can also result in secondary degeneration and malfunction in remote, but anatomically connected regions to the ischaemic lesion that can consequently alter brain networks. The concept of ‘diaschisis’ dates back over 130 years and can encompass alterations in energy metabolism, cerebral blood flow and neuronal activity that occur in anatomically distant regions from the focal lesion (Carrera and Tononi, 2014). The advent of novel imaging technologies, analyses and computational modelling have expanded the study of secondary changes to a systems level, connectome approach (or ‘connectional diaschisis’). These studies have shown that stroke-related neurological deficits and recovery depend not only on focal tissue damage, but also from local and distant changes in white matter tracts and alterations to network-wide processes (reviewed by Grefkes and Fink, 2014; Silasi and Murphy, 2014; Guggisberg *et al.*, 2019).

In the paper in *Brain Communications*, Schlemm *et al.* (2020) investigated alterations to the stroke connectome with advanced diffusion MRI in a prospective cohort of stroke patients with motor symptoms in the upper limb, aiming to relate changes in their global topology with clinical outcome. A novel aspect of this study was the longitudinal approach that enabled the evolution of topological properties of the structural connectome to be analysed from acute (∼1week), subacute (1 and 3 months) and chronic timepoints (1 year) and correlated with clinical course and lesion volume. The study benefitted from a relatively homogenous population of 30 patients with mild-to-moderate clinical deficits and modest lesions, predominantly in subcortical brain regions. For each time point, structural connectomes were reconstructed, and whole-hemisphere global network topology was quantified in terms of integration (the ability of the brain to integrate information from various sub-networks) and segregation (information about connectedness within separate segregated/specialized neural regions; see Lord *et al.*, 2017). There were three unique findings in the paper. Firstly, they found that the structural brain network after motor stroke evolves over time with a drift towards structural degeneration, impaired integration and greater segregation by 12 months post-stroke. These effects were in both hemispheres, but more pronounced in the hemisphere affected by stroke. Secondly, in the lesioned hemisphere, these changes were influenced by stroke volume, such that greater differences in network properties between acute and chronic phases were observed in patients with larger strokes. Because the disruptions of brain networks became more apparent at later timepoints, and were dependent of lesion volume, they potentially reflect the pathophysiological processes of secondary degeneration. Lastly, the magnitude of post-stroke change in structural connectivity was associated with residual symptom burden and motor impairment, and for most of these associations, this was independent of lesion volume. In contrast to the findings of others (reviewed in Guggisberg *et al.*, 2019), Schlemm *et al.* did not find evidence for increases in structural connectivity that have been suggested to indicate structural plasticity after stroke. Although this was a prospective study, no pre-stroke imaging was available for these patients. Therefore, future studies would benefit from pre-stroke imaging data to allow the interplay of pre-existing white matter alterations and structural network changes post-stroke to be examined.

It has long been known that recovery post-stroke whether motor or cognitive function is facilitated at different levels ranging from single cells to entire brain networks (Cramer, 2008). Various imaging and physiological methods and or combined multimodal protocols have been used to monitor functional recovery in stroke patients that are particularly important for stroke therapy trials. Transcranial magnetic stimulation (TMS) and the standard monitoring of motor evoked potential (MEP) has already been widely used to test the integrity of the corticospinal tract. In the study by Tscherpel and colleagues, a previously indicated approach (Auriat *et al.*, 2015) was rigorously undertaken by combining use of TMS-evoked EEG to measure function of local and network responses after stroke injury (Fig. 1). The study is of interest because the work-up protocols do not necessarily rely on intact corticospinal tract but can directly assess cortical excitability and activity incorporating cortical, subcortical, brainstem, spinal and peripheral processes. Evidently, their method allows generation of reliable readouts for individual patients with various types of stroke injury. To arrive at these conclusions, investigators recorded brain responses in the ipsilesional M1 region in 28 first-ever ischaemic stroke patients with unilateral mild-to-severe motor deficits comprising upper limb impairments, a proportion of whom were severely affected or plegic. They assessed them during an early subacute stage (<2 weeks post-stroke) and then reassessed 25 patients 3–6 months post-stroke. They found substantial alterations of the TMS-evoked EEG responses over the ipsilesional motor cortex for both local and remote effects. For the majority of patients, TMS elicited differentiated and sustained EEG responses with deflections sequentially involving both hemispheres and distant cortical regions similar to patterns found in healthy subjects. There were differences between healthy subjects and stroke patients for the prefrontal and motor region, but not for the parietal region. The highest overlaps of individual subject lesion maps were at the level of the basal ganglia, in the posterior limb of the internal capsule, and putamen indicating involvement of the thalamo-cortical fibres. Therefore, stroke-related responses detected via TMS–EEG are due to impaired connectivity rather than to primary dysfunction of M1 neurons. However, in the seven severely affected stroke patients, TMS triggered a simple and slow EEG response consistent with almost non-existent lasting motor function and disturbed corticospinal tracts as suggested by absent MEPs in these patients. The remarkable observation is that TMS–EEG can reveal differential motor cortex properties and discriminate between an otherwise homogenous group of stroke patients with severe damage. The measures of TMS-evoked EEG responses in the subacute stage were also closely related to initial motor deficits and degree of clinical recovery in chronic stages but surprisingly they did not find correlations with lesion volumes. While these findings on components of connectome including local cortical attributes and network dynamics play essential roles in pathological loss and motor recovery, TMS-evoked EEG (Sato *et al.*, 2015), permits a personalized assessment of each stroke patient to uncover more markers and target interventions for neuromodulation and rehabilitation.

**Figure 1:**
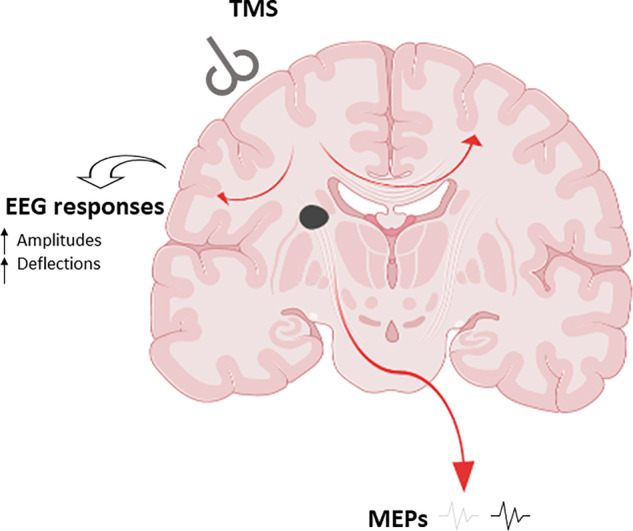
**TMS and post-stroke injury: coronal image at the level of the basal ganglia.** MEPs may be absent or weak after TMS but EEG responses reveal altered cortical activity post-stroke. When the patient does not present with MEPs, a combination of TMS-evoked EEG allows monitoring of cortical activity evoked by ipsilesional TMS. This can provide accurate prognosis and identify pathways for personalized interventions. Both high-resolution MRI and TMS–EEG approaches reveal impaired integration in the connectome in both hemispheres but less pronounced in the contralateral hemisphere affected by stroke.

Both studies by Schlemm *et al.* and Tscherpel *et al.*, highlight long-term changes in the structural or functional connectome after stroke, confirming that even discrete stroke lesions lead to widespread alterations in connectivity. Collectively, studying these alterations to brain network structure and its function will help us understand why some patients make better recovery than others, and may hold utility as a target for new treatment approaches and stratification of treatments, with prognosis and treatment adapted to the individual needs of each patient (Guggisberg *et al.*, 2019). Future studies would benefit from combining and analysing data from both functional and structural analyses. Compared with the wealth of studies examining brain networks involved in motor recovery after stroke, comparatively less known about alterations in the connectome associated with the recovery or onset of cognitive deficits after stroke. Therefore, more longitudinal studies in the same vein is required focused on cognitive changes. Furthermore, analogous to the widespread alterations in brain connectivity reported by connectome studies, the inflammatory response to stroke, originating in the focal lesion, is also reported to spread to distant brain regions in a delayed manner (Shi *et al.*, 2019), caused by increased levels of inflammatory cells and alterations to other glial cells in the brain. Integrating alterations to inflammatory glial cells, and their molecular changes with connectome studies will also be important for identifying new targets and understanding the mechanisms of connectome alterations post-stroke.
